# The negative effect of ANGPTL8 on HDL-mediated cholesterol efflux capacity

**DOI:** 10.1186/s12933-018-0785-x

**Published:** 2018-11-08

**Authors:** Mengdie Luo, Ziyu Zhang, Yani Peng, Shuai Wang, Daoquan Peng

**Affiliations:** 1 Department of Cardiovascular Medicine, The Second Xiangya Hospital, Central South University, No.139, Middle Renmin Road, Changsha, 410011 Hunan China; 2 Department of Metabolism & Endocrinology, The Second Xiangya Hospital, Central South University, Changsha, Hunan China

**Keywords:** ANGPTL8, Cholesterol efflux capacity, Coronary artery disease, Diabetes

## Abstract

**Background:**

It is well known that angiopoietin-like protein 8 (ANGPTL8) exerts its effects on lipid metabolism through the inhibition of lipoprotein lipase and subsequent elevation of plasma triglyceride. However, it is not clear whether ANGPTL8 could affect lipid metabolism via other pathways. The study was aimed to investigate the effects of ANGPTL8 on the function of high-density lipoprotein (HDL), which plays a protective role in atherosclerosis progression.

**Methods:**

Two hundred and ten subjects were recruited. Plasma ANGPTL8 was measured by enzyme-linked immunosorbent assays. Cholesterol efflux capacity was chosen as the biomarker of HDL function and measured via H^3^-cholesterol loading THP-1 cell models.

**Results:**

ANGPTL8 exhibited no significant difference between CAD group and nonCAD group, but ANGPTL8 in DM group was significantly higher than that in the nonDM group [568.3 (406.2–836.8) vs 458.2 (356.8–755.6), P = 0.023]. Compared to controls, subjects in CAD group and DM group exhibited significantly lower cholesterol efflux capacity [CAD: 14.58 ± 2.06 vs 12.51 ± 2.83%, P < 0.0001; DM: 13.62 ± 2.57 vs 12.34 ± 3.16%, P = 0.0099]. ANGPTL8 was inversely correlated with cholesterol efflux capacity (r = − 0.188, P < 0.01). Regression analysis revealed that plasma ANGPTL8 was an independent contributor to cholesterol efflux capacity (standardized β = − 0.143, P = 0.023).

**Conclusion:**

ANGPTL8 presents a negative effect on HDL-mediated cholesterol efflux capacity.

**Electronic supplementary material:**

The online version of this article (10.1186/s12933-018-0785-x) contains supplementary material, which is available to authorized users.

## Background

Many epidemiological studies revealed that high-density lipoprotein cholesterol (HDL-C) level was inversely correlated with CAD incidence and presented HDL-C as a robust biomarker of cardiovascular risk [1, 2]. However, clinical trials [3, 4] and Mendelian randomization studies [5] failed to prove the causative association of HDL-C and clinical benefits. Due to the disappointing outcomes of raising HDL-C and the heterogeneity of components across HDL particles, HDL functionality emerged as a more intriguing target. Among multiple atheroprotective effects of HDL, its participation in reverse cholesterol transport (RCT) was demonstrated to be the most important [6]. Cholesterol efflux from peripheral tissue and cells, the initial step of RCT, presented a strongly inverse association with carotid intima-media thickness and CAD likelihood independent of HDL-C level [7]. Our previous study in patients with chronic kidney diseases also confirmed the reverse correlation between cholesterol efflux and carotid intima-media thickness [8]. A prospective cohort study revealed that HDL-mediated cholesterol efflux capacity, instead of HDL-C level, was an independent predictor of CAD incidence [9]. Therefore, it may help the development of therapeutic interventions to unravel the molecular determinants of HDL-mediated cholesterol efflux capacity and improve HDL function [10].

The angiopoietin-like proteins (ANGPTL1-8) are secreted glycoproteins sharing common structures but exerting distinct physiological effects [11]. ANGPTL8, also referred to as betatrophin, lipasin, refeeding-induced in fat and liver (RIFL) and hepatocellular carcinoma-associated protein (TD26), was found to be a novel player in lipid metabolism [12]. ANGPTL8, together with ANGPTL3 and ANGPTL4, could regulate triglyceride metabolism by inhibiting the activity of lipoprotein lipase (LPL) [13, 14], the rate-limiting enzyme for triglyceride hydrolysis and plasma triglyceride clearance [15]. Genome-wide association studies (GWAS) in American Indians and Mexican Americans identified that ANGPTL8 variant was associated with HDL-C level [16]. A cross-sectional study targeted at Japanese subjects also found that increased ANGPTL8 concentration was inversely correlated with circulating HDL-C [17]. Another study compared the change in serum ANGPTL8 concentration after bariatric surgery and found that the change in ANGPTL8 concentration was positively correlated with change in HDL-C concentration [18]. Although many studies have studied the relationship between ANGPTL8 and HDL-C, however, it is still unclear whether ANGPTL8 could affect HDL function. As mentioned above, HDL-mediated cholesterol efflux capacity is a significant surrogate of HDL function. Therefore, in the present study, we conducted an observational study to examine the relationship between ANGPTL8 and HDL-mediated cholesterol efflux capacity.

## Methods

### Subjects

We recruited 120 CAD subjects and 90 nonCAD subjects from the Department of Cardiovascular Medicine of Second Xiangya Hospital, Central South University. In this study, CAD meant acute coronary syndrome (ACS) and DM meant type 2 diabetes mellitus. ACS included ST-segment elevated myocardial infarction (STEMI), non ST-segment elevated myocardial infarction (NSTEMI) and unstable angina. ACS diagnosis was based on the clinical symptoms and signs, ischemic electrocardiographic abnormalities, and coronary angiography showing ≥ 50% stenosis in at least one main coronary artery. The exclusion criteria included: a history of renal failure, chronic hepatic diseases, high fever, or bacterial/viral infection, autoimmune disease, arthritis, malignancies, severe diabetes and hypertension, and other severe medical illnesses. Diagnosis of T2DM was based on one of the following criteria: fasting plasma glucose level of ≥ 126 mg/dL (≥ 7.0 mmol/L), random plasma glucose of ≥ 200 mg/dL (≥ 11.1 mmol/L), or plasma glucose of ≥ 200 mg/dL (≥ 11.1 mmol/L) after administration of 75 g oral glucose tolerance test (OGTT). The inclusion criteria were (i) being diagnosed with T2DM for more than 1 year, (ii) no antibiotics or steroidal and nonsteroidal anti-inflammatory medications being used during the last 3 weeks, and (iii) not treated with immunosuppressive chemotherapy, no current acute illness present, no professional periodontal treatment received during the last 6 months, and no ongoing pregnancy or lactation. Informed consent was obtained from each patient.

### Clinical and biochemical measurements

Patient information, including age, gender, smoking and drinking history, and statin therapy history, was recorded. The details of anthropometric measurements (weight, height, body mass index) were assessed after overnight fasting for at least 10 h. Peripheral blood samples were obtained from patients’ brachial veins. Subjects fasted for at least 10 h before blood collection and then blood routine, urine routine, concentrations of lipid parameters, including total cholesterol (TC), triglyceride (TG), low-density lipoprotein cholesterol (LDL-C), HDL-C, apoAI, apoB, free fatty acid (FFA), were evaluated via standard laboratory procedures. Concentrations of high-sensitivity C-reactive protein (hsCRP) were measured with a latex particle, enhanced immunoturbidimetric assay. For the subsequent experiments, fresh plasma was obtained by centrifugation at 3000r/min at 4 °C for 10 min. The plasma was aliquoted and stored at − 80 °C freezer until analysis.

### Measurement of plasma ANGPTL8

Plasma ANGPTL8 concentration was measured with commercially available ELISA kits (ANGPTL8: EIAAB, E11644H, Wuhan, China). All the measurement of plasma ANGPTL8 was performed in duplicate for each sample. The coefficient of variation for intra- and inter-assay variation was < 6% and < 9%, respectively.

### ApoB-depleted plasma preparation

According to previous reported procedures [19], 540 μL heparin sodium solution (280 mg/mL, Aladdin, H104201) and 10 mL manganese chloride solution (1.06 mol/L, Aladdin, M112542) were mixed. 100 μL mixed solution was added to 1 mL plasma, incubated for 30 min at 4 °C, followed by centrifugation at 1500*g* for 30 min. The supernatant was collected. If supernatant was still turbid (especially samples from patients with hypertriglyceridemia), plasma was centrifuged at 12000*g* for 10 min again. The previous study revealed that heparin sodium/manganese chloride precipitation had no effects on HDL size as well as cholesterol efflux measurement [20], and therefore this method was chosen to prepare apoB-depleted plasma in the study.

### Cholesterol efflux capacity measurement

Cholesterol efflux experiments were performed according to established procedures [21, 22]. THP-1 human monocytes (ATCC) were grown in RPMI1640 medium (Gibco, 22400089), supplemented with 10% heat-inactivated FBS, 1% penicillin/streptomycin until differentiation into macrophages by the addition of phorbol myristate acetate (100 ng/mL, Sigma, P1585). Subsequently, differentiated THP-1 macrophages were loaded with 50 μg/mL acetylated LDL (Peking Union-Biology Co. Ltd) and 1 μCi/mL [3H] cholesterol for 24 h. Macrophages were then washed twice with PBS (Gibco, 10010023) and equilibrated for 24 h in RPMI1640 medium containing 2% bovine serum albumin. Cells were then washed with PBS again and apoB-depleted plasma from individual patients was diluted in medium (2.5%, vol/vol). After 16 h, the supernatant was collected and centrifuged to remove cellular debris. The cells were washed twice with PBS, and then incubated for at least 30 min at room temperature with 0.1 mol/L NaOH solution. The radioactivity within the supernatant and cells was determined by liquid scintillation counting. Wells incubated with RPMI1640 but without added apoB-depleted plasma were used as blanks, and these values were subtracted from the respective experimental values. Efflux is given as the percentage of counts recovered from the medium in relation to the total counts present on the plate (sum of medium and cells). All efflux experiments were performed in duplicate for each sample. The coefficient of variation for intra- and inter-assay variation was < 4% and < 8%, respectively.

### Statistical analysis

Statistical analysis was performed with Statistical Package for Social Sciences version 22.0 and plots were made with GraphPad Prism V.6.0 (GraphPad Software, Inc, La Jolla, California, USA). Clinical data are expressed as mean ± standard deviation (normally distributed continuous data) or median with interquartile range (skewed distributed continuous data). Comparisons between categorical data were performed with Chi-Squared tests, while continuous variables were assessed by unpaired t test (for normal distribution) or nonparametric test (for skewed distribution). To evaluate the associations between variables, Pearson correlation analysis was used. Stepwise multiple linear regression analysis was performed to determine the variables with independent significant association with cholesterol efflux capacity. These variables included all potential ones that might have a significant relationship with cholesterol efflux capacity in univariate analyses. In the correlation and regression analysis, logarithmic-transformed values were used for the variables skewed distributed. A two-tailed P value < 0.05 was considered statistically significant.

## Results

### Characteristics of subjects

Demographic and biochemical characteristics of participants were shown in Table 1. The study included 210 unrelated individuals, 60.9% of the participants were male and the mean age was 64.76 years. Compared to nonCAD controls, CAD subjects had significantly higher free fatty acid (FFA) and statin use, while other biomarkers are significantly lower, including total cholesterol (TC), high-density lipoprotein cholesterol (HDL-C), low-density lipoprotein cholesterol (LDL-C) and apolipoprotein AI (apoAI). When dividing the subjects into DM group and nonDM group, DM subjects had significantly higher body mass index (BMI) and FFA level.

**Table 1:** Baseline characteristics of all the subjects

Variables	CAD (n = 120)	nonCAD (n = 90)	P	DM(n = 37)	nonDM (n = 173)	P
Male (%)	63.6	57.3	NS	59.8	61.3	NS
Age (years)	63.96 ± 7.85	63.09 ± 8.25	NS	64.32 ± 7.36	63.41 ± 8.18	NS
BMI (kg/m^2^)	24.17 ± 3.92	24.42 ± 3.02	NS	25.47 ± 3.40	24.00 ± 3.54	0.024
TG (mg/dl)	128.43 ± 71.74	129.32 ± 79.72	NS	147.92 ± 91.23	124.00 ± 69.97	NS
TC (mg/dl)	145.40 ± 35.96	158.16 ± 39.83	0.005	146.95 ± 40.22	151.97 ± 37.51	NS
HDL-C (mg/dl)	40.60 ± 10.05	44.08 ± 11.21	0.028	39.06 ± 9.28	42.92 ± 10.83	NS
LDL-C (mg/dl)	88.16 ± 30.16	97.06 ± 31.32	0.021	88.17 ± 31.32	92.81 ± 30.94	NS
apoAI (g/L)	1.08 ± 0.20	1.17 ± 0.25	0.008	1.08 ± 0.20	1.12 ± 0.23	NS
apoB (g/L)	0.85 ± 0.25	0.89 ± 0.26	NS	0.89 ± 0.28	0.86 ± 0.25	NS
hsCRP (mg/L)	4.93 (1.26–13.44)	2.36 (1.17–7.00)	NS	5.12 (1.62–8.28)	3.23 (1.18–11.51)	NS
FFA (mmol/L)	0.52 ± 0.29	0.44 ± 0.27	0.047	0.57 ± 0.29	0.46 ± 0.28	0.045
Statin (%)	55.5	3.8	< 0.0001	35.1	32.9	NS
Smoking (%)	46.4	30.5	0.036	37.8	40.0	NS
HF (%)	72.7	42.7	< 0.0001	72.9	57.2	NS

Values are expressed as mean ± SD or median (interquartile range). *CAD* coronary artery disease, *DM* diabetes mellitus, *BMI* body mass index, *TG* triglyceride, *TC* total cholesterol, *HDL-C* high-density lipoprotein cholesterol, *LDL-C* low-density lipoprotein cholesterol, *apoAI* apolipoprotein AI, *apoB* apolipoprotein B, *hsCRP* high sensitivity C reactive protein, *FFA* free fatty acid, *HF* heart failure

### ANGPTL8 concentration in subjects

**Fig. 1:**
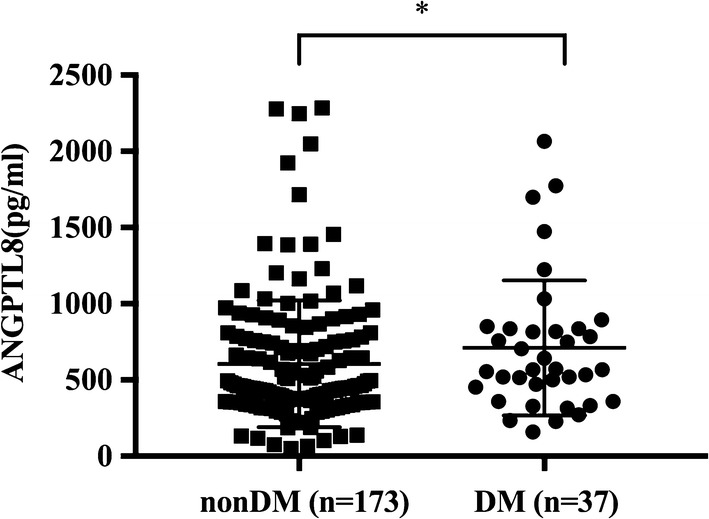
ANGPTL8 from nonDM (n = 173) and DM (n = 37) patients. Data are expressed as median ± interquartile range. DM indicates diabetes mellitus. *P < 0.05

**Fig. 2:**
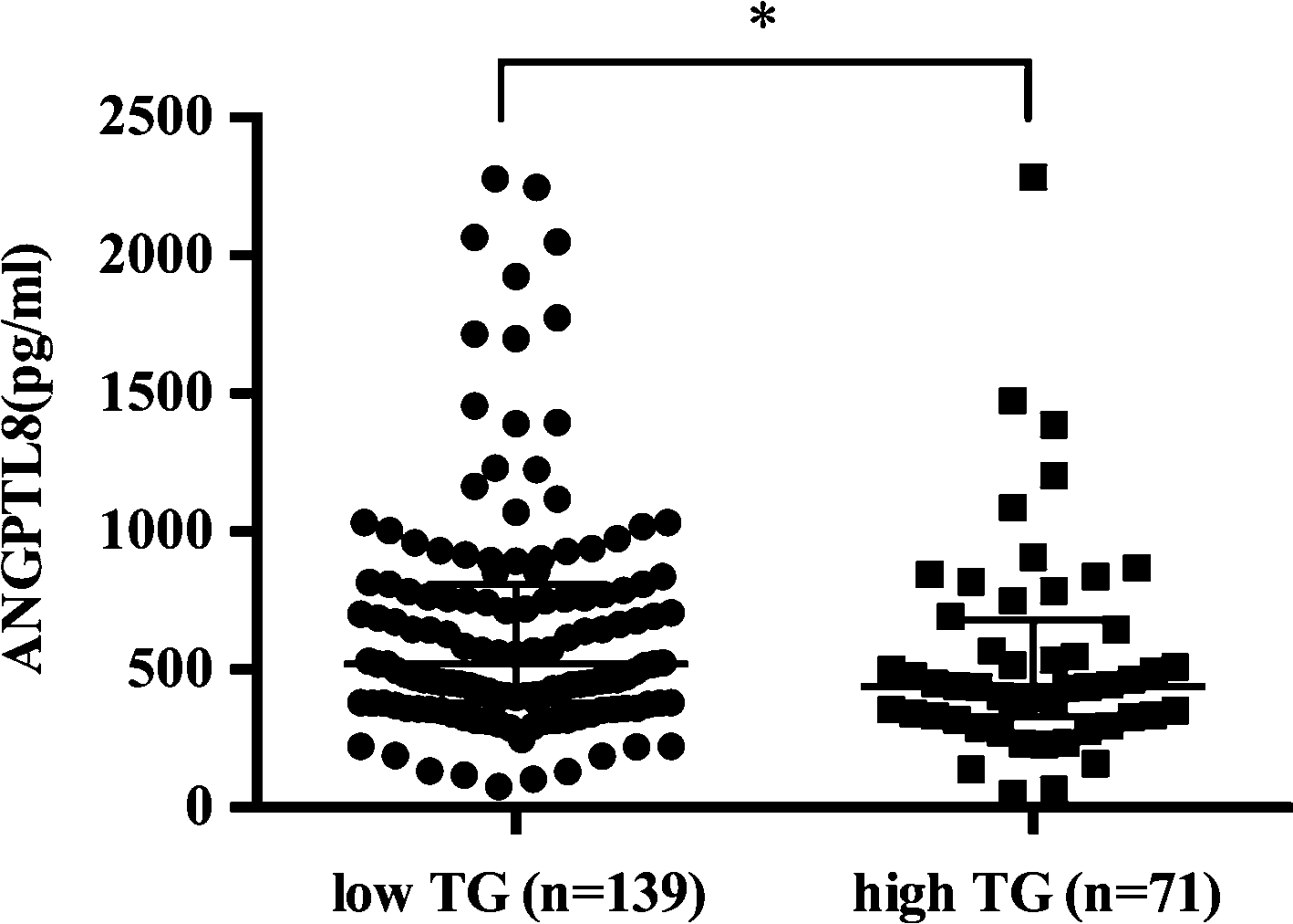
ANGPTL8 from low triglyceride group (n = 139) and high triglyceride group (n = 71) patients. Data are expressed as median ± interquartile range. TG indicates triglyceride. *P < 0.05

ANGPTL8 concentration between CAD and nonCAD groups was not significantly different [504.5 (364.4–774.4) vs 452.8 (354.8–800.1), P = 0.736]. However, ANGPTL8 level in those with DM was significantly higher than those without DM [568.3 (406.2–836.8) vs 458.2 (356.8–755.6), P = 0.023, Fig. 1]. ANGPTL8 concentration in male subjects was also significantly higher than that in female subjects [535.8 (398.8–836.9) vs 430.3 (338.0–692.9), P = 0.007]. In addition, when subjects were divided into low triglyceride group (defined as < 150 mg/dL) and high triglyceride group (defined as ≥ 150 mg/dL) according to previous report [23, 24], ANGPTL8 presented significantly higher level in low triglyceride group than in high triglyceride group [519.9 (377.6–809.0) vs 438.7 (329.2–681.0), P = 0.038, Fig. 2]. However, no significant difference of ANGPTL8 concentration was found between statin group and non-statin group [463.2 (356.6–745.3) vs 515.3 (377.4–831.3), P = 0.139].

### Cholesterol efflux capacity in subjects

**Fig. 3:**
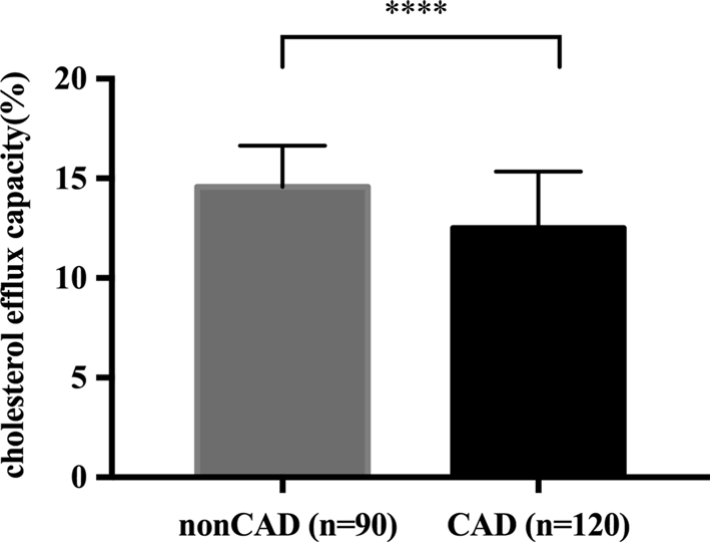
Cholesterol efflux capacity in nonCAD (n = 90) and CAD (n = 120) patients. Data are expressed as mean ± SD. CAD indicates coronary artery disease. ****P < 0.0001

**Fig. 4:**
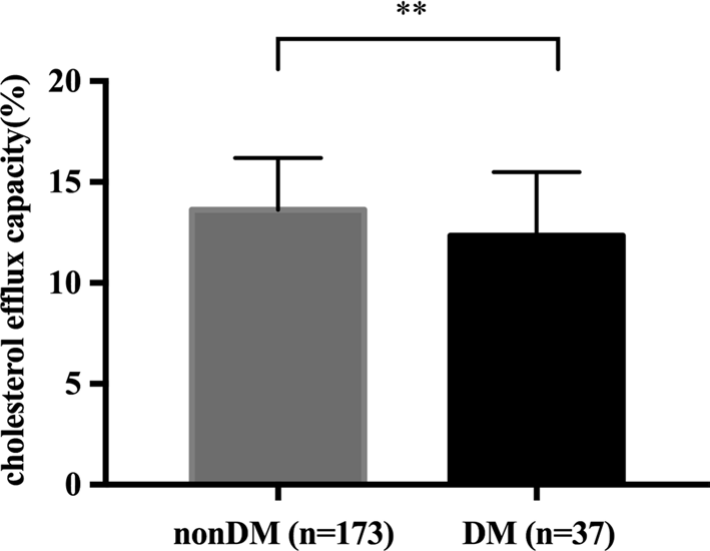
Cholesterol efflux capacity in nonDM (n = 173) and DM (n = 37) patients. DM indicates diabetes mellitus. Data are expressed as mean ± SD. **P < 0.01

Cholesterol efflux capacity in CAD subjects and DM subjects was significantly lower compared to nonCAD subjects and nonDM subjects (CAD: 12.51 ± 2.83 vs 14.58 ± 2.06%, P < 0.0001, Fig. 3; DM: 12.34 ± 3.16 vs 13.62 ± 2.57%, P = 0.0099, Fig. 4).

### Correlation analysis of clinical variables in subjects

**Fig. 5:**
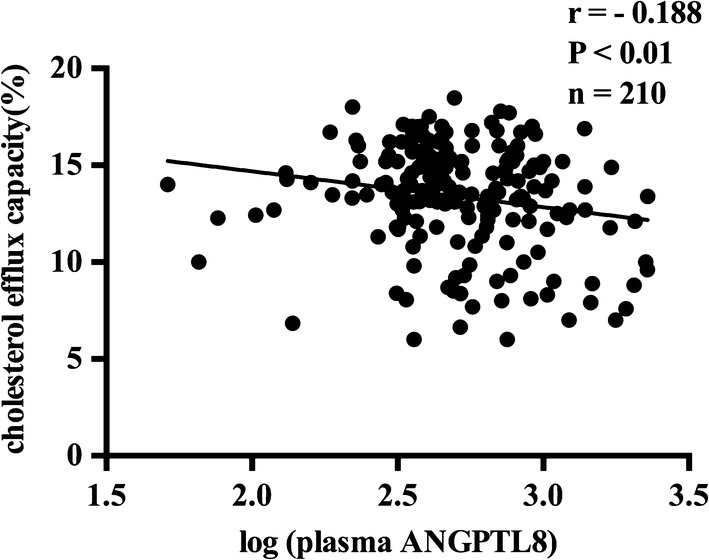
The correlation between plasma ANGPTL8 (log-transformed) and cholesterol efflux capacity in all the subjects

To investigate variables associated with ANGPTL8 and cholesterol efflux capacity, correlation analysis was performed. In all the participants, plasma ANGPTL8 (log-transformed) presented a significant inverse relationship with cholesterol efflux capacity (r = − 0.188, P < 0.01, n = 210, Fig. 5). Plasma ANGPTL8 (log-transformed) is also inversely correlated with HDL-mediated cholesterol efflux capacity in CAD group (r = − 0.247, P < 0.01, n = 120, Additional file 1: Fig. S1) and nonDM group (r = − 0.164, P < 0.05, n = 173, Additional file 2: Fig. S2). At the same time, plasma ANGPTL8 (log-transformed) and cholesterol efflux capacity showed a significant correlation with other biomarkers (see Table 2).

**Table 2:** Pearson’s correlations between clinical variables and log-transformed ANGPTL8 as well as cholesterol efflux capacity in all the subjects

	ANGPTL8 (log-transformed)		Cholesterol efflux capacity	
	r	P	r	P
Age	0.250	< 0.001	− 0.021	NS
HDL-C	− 0.075	NS	0.402	< 0.0001
LDL-C	− 0.154	0.034	0.216	< 0.01
TC	− 0.184	0.011	0.266	< 0.0001
TG	− 0.099	NS	0.130	NS
ApoAI	− 0.124	NS	0.527	< 0.0001
BUN	0.347	<0.0001	− 0.125	NS
UA	0.374	< 0.0001	− 0.251	< 0.001
CR	0.412	< 0.0001	− 0.126	NS
hsCRP (Log-transformed)	0.098	NS	− 0.244	< 0.001

*ANGPTL8* angiopoietin-like protein 8, *HDL-C* high density lipoprotein-cholesterol, *LDL-C* low density lipoprotein-cholesterol, *TC* total cholesterol, *TG* triglyceride, *apoAI* apolipoprotein AI, *BUN* blood urea nitrogen, *UA* uric acid, *CR* creatinine, *hsCRP* high-sensitivity C reactive protein

### Multivariate analysis for the associations of clinical variables to cholesterol efflux capacity

In order to determine the independent contributors to the cholesterol efflux capacity, stepwise multiple regression models were fitted after adjustment for different variables. Log-transformed values were used for the variables skewed distributed, including ANGPTL8 and hsCRP. The regression analysis revealed that ANGPTL8, apoAI and statin use were independent risk factors to the cholesterol efflux capacity (Table 3).

**Table 3:** Stepwise multiple regression analysis detecting independent contributors to HDL-mediated cholesterol efflux capacity in all the subjects

Factors	β	Standardized β	P
apoAI	0.057	0.481	< 0.001
ANGPTL8 (log-transformed)	− 0.014	− 0.143	0.023
hsCRP (log-transformed)	− 0.002	− 0.048	0.482
LDL-C	0.001	0.028	0.675
Statin use	− 0.008	− 0.140	0.027

R square: 0.316, P < 0.001

## Discussion

In this study, we found that ANGPTL8 showed no significant difference between CAD and nonCAD groups, while ANGPTL8 in DM group was significantly higher compared to nonDM controls. Besides, ANGPTL8 was inversely correlated with HDL-mediated cholesterol efflux capacity. Stepwise multiple regression analysis revealed that ANGPTL8 was an independent contributor to HDL-mediated cholesterol efflux capacity.

### ANGPTL8 and blood lipids

ANGPTL8 has been reported to present significant but contradictive relationship with various blood lipid markers. Genomic studies showed that ANGPTL8 gene variant was associated with HDL-C variant [16, 25]. The relationship between ANGPTL8 and triglyceride has also been deeply excavated. A recently published paper reported that ANGPTL8 knockout mediated by CRISPR-Cas9 system led to reduced plasma triglyceride levels [26], which verified conclusions from animal experiments—ANGPTL8 was found to inhibit LPL activity and elevate plasma triglyceride partly via ANGPTL3 activation after feeding [14]. A prospective cohort study conducted in Korean children revealed that baseline ANGPTL8 was associated with future changes in triglyceride levels [27]. However, in our research, ANGPTL8 presented no correlation with triglyceride and HDL-C level, even though ANGPTL8 was inversely associated with LDL-C and total cholesterol. Results from epidemiological studies are inconsistent [16–18, 24]. The relationship between ANGPTL8 and blood lipid markers might be confounded by sample selection and age, which is positively correlated with ANGPTL8 in our study and other literature [28]. Besides, a study focused on the effects of vitamin D on the relationship between ANGPTL8 and cardiometabolic risk factors has found that the association varied in different vitamin D status [23]. In our study, patients were not stratified by vitamin D status, and it might be a potential reason for the discrepancies among studies.

Of note, ANGPTL8 presented a strongly positive relationship with biomarkers of renal function in our study, including BUN, UA and CR. A previous research studied the association of ANGPTL8 and diabetic nephropathy and found that ANGPTL8 was predictive of the progression of diabetic nephropathy [29]. Abnormal ANGPTL8 elevation may lead to dysregulated lipid metabolism in the kidney, which offers a possible explanation for the role of ANGPTL8 in the development of diabetic nephropathy.

### ANGPTL8 and HDL function

HDL-mediated cholesterol efflux capacity was inversely associated with cardiovascular risk independent of HDL-C concentration [7, 9, 30]. The ability of HDL to mediate cholesterol efflux from lipid-laden peripheral macrophages is the crucial part of HDL atheroprotective function. In inflammatory conditions, such as coronary artery disease, the lipid and protein composition of HDL can also be altered, a process which is called HDL remodeling. HDL inflammatory remodeling could impair cholesterol efflux. Our previous study found that apoAI modification mediated by myeloperoxidase oxidation could impair cholesterol efflux capacity [22]. Increased serum amyloid A(SAA) in HDL particles could result in HDL trapping in the extracellular matrix and impair HDL-mediate cholesterol efflux [31, 32].

Our previous study revealed that apolipoprotein CIII(apoCIII) enrichment was inversely correlated with HDL-mediated cholesterol efflux [33]. However, the relationship between apoCIII and HDL-mediated cholesterol efflux was independent of inflammatory biomarker such as hsCRP level, therefore, we hypothesized that apoCIII might play an atherogenic role independent of its pro-inflammatory effects. In this study, we found that ANGPTL8 concentration was an independent contributor to cholesterol efflux capacity after controlling for hsCRP. A previous study showed that ANGPTL8 was increased in metabolic syndrome patients and presented a strongly positive correlation with hsCRP [28], implying a potential role of ANGPTL8 in inflammation. However, Zhang et al. [34] found that ANGPTL8 functioned as a negative regulator in nuclear factor-κB activation triggered by tumor necrosis factor α. Therefore, it seems invalid that ANGPTL8 may impair HDL-mediated cholesterol efflux via inflammation-related pathways.

ApoAI removes cholesterol from peripheral cells via membrane transporter ATP-binding cassette A1(ABCA1) to form nascent HDL particles [35]. Increased unsaturated fatty acids can enhance ABCA1 degradation and reduce membrane ABCA1 content, leading to impaired cholesterol efflux mediated by ABCA1 pathway [36]. Dang et al. [37] found that ANGPTL8 overexpression could dramatically elevate non-esterified fatty acids content. Based on these findings, we hypothesized that ANGPTL8 might impair HDL-mediated cholesterol efflux via the positive effects on unsaturated fatty acids and subsequent ABCA1 decrease.

ANGPTL8 could promote ANGPTL3 cleavage and form a complex with the cleaved N-terminal of ANGPTL3 [14]. The complex was demonstrated to inhibit LPL activity and modulate lipid metabolism. The close relationship between ANGPTL3 and ANGPTL8 casts a shadow over the studies which are designed to explore the independent effects of ANGPTL3 or ANGPTL8 on HDL function. Zhao et al. [38] studied the association between ANGPTL3 and HDL-mediated cholesterol efflux on T2DM subjects. Their findings showed that plasma ANGPTL3 increase was positively associated with apoAI content in HDL particles from female diabetic patients, however, plasma ANGPTL3 increase presented a weak and insignificant inverse relationship with change of HDL-mediated cholesterol efflux [38]. It is highly possible that ANGPTL3 and ANGPTL8 could reside on HDL particles and those ANGPTL3 and ANGPTL8 molecules residing on HDL particles were the real contributors to HDL functional change.

Although HDL-mediated cholesterol efflux was thought to play an important role in atheroprotection and found to be inversely associated with atherosclerosis incidence, however, a previous study reported that increased cholesterol efflux to apoB-depleted serum was positively associated with incident cardiovascular events [39]. The paradoxical findings implicated the limitations of cholesterol efflux assay. Excessive cholesterol needs to be removed from peripheral macrophages to apoAI or HDL and return to the liver for biliary excretion. In vitro cholesterol efflux assay cannot fully demonstrate the whole reverse cholesterol transport pathway and the validity should be carefully interpreted.

## Conclusions

Our study presented that ANGPTL8 exerted a negative effect on HDL-mediated cholesterol efflux capacity, which provided evidence for the role of ANGPTL8 in HDL dysfunction and a possible explanation for the atherogenic effects of ANGPTL8. However, further research is warranted to elucidate the underlying mechanism of ANGPTL8 effects.

## Additional files

**Additional file 1: Figure S1.** The correlation between plasma ANGPTL8 (log-transformed) and cholesterol efflux capacity in CAD subjects.

**Additional file 2: Figure S2.** The correlation between plasma ANGPTL8 (log-transformed) and cholesterol efflux capacity in nonDM subjects.

## Abbreviations

CAD: coronary artery disease
DM: diabetes mellitus
ANGPTL8: angiopoietin-like protein 8
HDL: high density lipoprotein
LDL: low density lipoprotein
TG: triglyceride
TC: total cholesterol
BMI: body mass index
BUN: blood urea nitrogen
UA: uric acid
CR: creatinine
hsCRP: high-sensitivity C reactive protein
apoAI: apolipoprotein AI
apoB: apolipoprotein B
FFA: free fatty acid
