# Supplementary figures and images for: The negative effect of ANGPTL8 on HDL-mediated cholesterol efflux capacity
Source: Cardiovasc Diabetol. 2018 Nov 8;17:142. doi: 10.1186/s12933-018-0785-x (PMC6223079; doi:10.1186/s12933-018-0785-x)

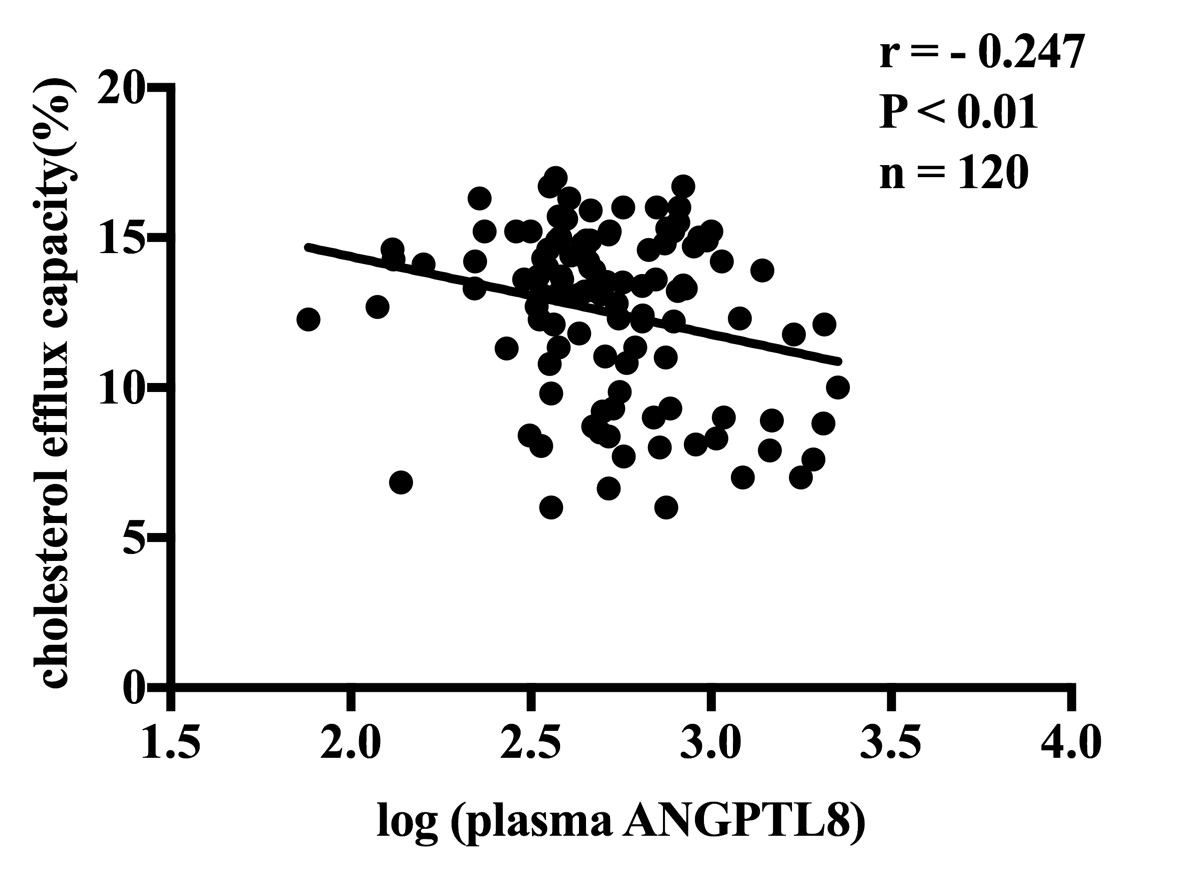

Supplement: Supplementary file 1 — Additional file 1: Figure S1. The correlation between plasma ANGPTL8 (log-transformed) and cholesterol efflux capacity in CAD subjects. [file 12933_2018_785_MOESM1_ESM.tiff]

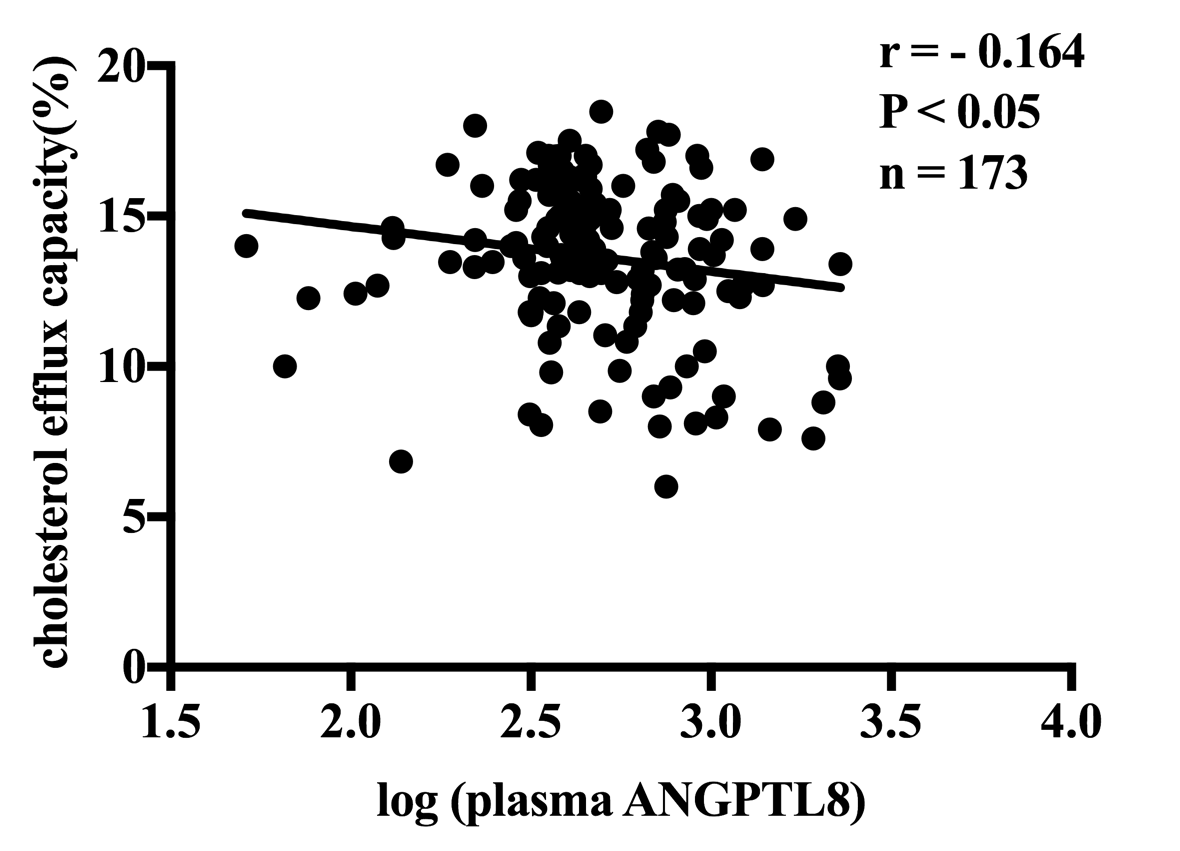

Supplement: Supplementary file 2 — Additional file 2: Figure S2. The correlation between plasma ANGPTL8 (log-transformed) and cholesterol efflux capacity in nonDM subjects. [file 12933_2018_785_MOESM2_ESM.tiff]
